# Evaluation of an adaptive, multimodal intervention to reduce postoperative infections following cesarean delivery in Ethiopia: study protocol of the CLEAN-CS cluster-randomized stepped wedge interventional trial

**DOI:** 10.1186/s13063-022-06500-9

**Published:** 2022-08-19

**Authors:** Tihitena Negussie Mammo, Mekdes Daba Feyssa, Sara Taye Haile, Tesfaneh Fikre, Matiyas Asrat Shiferaw, Habtamu Woldeamanuel, Fikremelekot Temesgen, Natnael Gebeyehu, Nichole Starr, Katie Fernandez, Natalie Henrich, Senait Bitew Alemu, Kate Miller, Thomas G. Weiser

**Affiliations:** 1 Department of Surgery, Addis Ababa University; Lifebox Foundation, Addis Ababa, Ethiopia; 2 Department of Obstetrics & Gynecology, St. Paul’s Hospital Millennium Medical College; Ethiopian Society of Obstetricians and Gynecologists; Center for International Reproductive Health Training, Addis Ababa, Ethiopia; 3 Lifebox Foundation, Addis Ababa, Ethiopia; 4 Ethiopian Society of Obstetricians and Gynecologists, Addis Ababa, Ethiopia; 5 Department of Obstetrics & Gynecology, St. Paul’s Hospital Millennium Medical College; Lifebox Foundation, Addis Ababa, Ethiopia; 6 Lifebox Foundation, Addis Ababa, Ethiopia; 7 Department of Obstetrics, Addis Ababa University; Ethiopian Society of Obstetricians and Gynecologists, Addis Ababa, Ethiopia; 8 Department of Surgery, University of California San Francisco, San Francisco, CA USA; 9 Lifebox Foundation, London, UK; 10 Ariadne Labs, Harvard T.H. Chan School of Public Health, Boston, MA USA; 11 Ariadne Labs, Harvard T.H. Chan School of Public Health, MB Boston, USA; 12 Department of Surgery, Stanford University School of Medicine, Stanford, CA USA

## Abstract

**Background:**

We previously developed and pilot tested Clean Cut, a program to prevent postoperative infections by improving compliance with the WHO Surgical Safety Checklist (SSC) and strengthening adherence to infection control practices. This protocol describes the *C*heck*L*ist *E*xpansion for *A*ntisepsis and i*N*fection Control in *C*esarean *S*ection (CLEAN–CS) trial evaluating our program’s ability to reduce infections following CS and other obstetric and gynecological operations in Ethiopia.

**Methods/design:**

CLEAN-CS is a cluster-randomized stepped wedge interventional trial with five clusters (two hospitals per cluster). It aims to assess the impact of Clean Cut on six critical perioperative infection prevention standards including antiseptic practices, antibiotic administration, and routine SCC use. The trial involves baseline data collection followed by Clean Cut training and implementation in each cluster in randomized order. The intervention consists of (1) modifying and implementing the SSC to fit local practices, (2) process mapping each standard, (3) coupling data and processes with site-specific action plans for improvement, and (4) targeted training focused on process gaps. The primary outcome is 30-day CS infection rates; secondary outcomes include other patient-level complications and compliance with standards. Assuming baseline SSI incidence of 12%, an effect size of 25% absolute reduction, and the ability to recruit 80–90 patients per cluster per month, we require a sample of 8100 patients for significance. We will report our study according to CONSORT.

**Discussion:**

A cluster-randomized stepped wedge design is well-suited for evaluating this type of surgical safety program. The targeted standards are not in doubt, yet compliance is frequently difficult. Solutions are available and may be recognized by individuals, but teams dedicated to improvement are often lacking. Clean Cut was successfully piloted but requires a more rigorous methodological assessment. We seek to understand the qualities, characteristics, and resources needed to implement the program, the magnitude of effect on processes and outcomes, and to what degree it can enhance compliance with care standards. Challenges include a fraught social and political environment, pandemic travel restrictions, and a limited budget.

**Trial registration:**

ClinicalTrials.gov NCT04812522 (registered on March 23, 2021); Pan-African Clinical Trials Registry PACTR202108717887402 (registered on August 24, 2021).

**Supplementary Information:**

The online version contains supplementary material available at 10.1186/s13063-022-06500-9.

## Background

Cesarean delivery, or C-section (CS), is the single most common major surgical procedure performed worldwide. It accounts for 7% of all operations [1], and in low resource settings CS can comprise up to 50% or more of the total volume of operations performed in a surgically capable facility. The World Health Organization recommends national CS rates of between 10 and 15% to save lives and improve maternal and neonatal outcomes. Population-based work indicates that CS rates of up to 19% are demonstrably related to improved maternal and neonatal survival [2]. However, complications are common, and gynecological and obstetric surgical interventions are associated with high rates of morbidity. In low resource settings, complication rates are particularly high.

In Ethiopia, maternal mortality is 401 per 100,000 live births and neonatal mortality 30 per 1000 live births [3]. While CS accounts for 30–50% of all operations performed within the country [4], a finding consistent with other similarly resourced countries, the national CS rate per live birth was 1.9% in 2016; however, rates are highly variable by region. Approximately 10.6% of births occur by CS in urban areas, compared to 0.9% of CS births in rural areas: Addis Ababa has a CS rate of 21.4%, Harari district has a rate of 9.0%, Dire Dawa 5.3%; all other regions have rates below 3% [5]. Furthermore, distribution of capabilities is clearly inconsistent, complications are common, and gynecological and obstetric surgical interventions are associated with high rates of morbidity.

Infections and complications following CS are estimated to cause 15% of maternal deaths in the country [6], and the overall surgical site infection (SSI) rate following CS is estimated at 9% [7]. Failure to administer preoperative antibiotics has been highlighted as a particular improvement opportunity [8]. As part of the quality improvement work focused on surgery and anesthesia, the Ethiopian Federal Ministry of Health (FMOH) launched its SALTS program – Saving Lives Through Safe Surgery [9]. Lifebox, a charity devoted to improving surgical and anesthesia safety, commenced a program in conjunction with these efforts to improve compliance with the WHO Surgical Safety Checklist and improve adherence to critical standards of perioperative infection prevention. This initiative, called Clean Cut, is an adaptive, multimodal surgical infection prevention program that integrates perioperative process improvement and patient outcomes measurement using process mapping, training and improved management practices, and compliance with critical standards of surgical antisepsis [10]. The program was the result of a joint collaboration between the FMOH, the Surgical Society of Ethiopia, and Lifebox.

Clean Cut was successfully piloted in five surgical departments in Ethiopia and reduced the relative risk of infection by 35% using a prospective pre/post pragmatic assessment [11]. The approach was pragmatic and used a pre/post assessment to evaluate its effect. It established a data collection mechanism to assess compliance with best practices and undertook a process mapping exercise to identify problems. Each facility team subsequently compared these data and process maps and identified interventions that could improve compliance based on local knowledge and experience. A weakness of this approach was an inability to distinguish the effect of data collection alone as a driver of improvement (the Hawthorne Effect) or the various elements and characteristics that promoted or prevented improvements. Teams also undertook implementation when they felt prepared to do so, which varied from site to site. We have adapted this intervention specifically for obstetric and gynecological operations and will implement it in ten maternity hospitals/departments using a cluster-randomized stepped wedge trial design. The protocol presented here follows the SPIRIT 2013 guidelines for protocol reporting and includes a SPIRIT checklist (Additional file 1) [12]. The objective is to assess the impact of this program in reducing infections and other complications for women undergoing CS and other obstetric and gynecologic operations. We seek to understand the qualities, characteristics, and resources needed to implement the program, the magnitude of the effect on process compliance and resultant outcomes of care, and the effect the specific activities of process improvement have on compliance with critical standards of perioperative infection prevention and control [13].

## Methods and design

### Trial design

This trial is a cluster-randomized stepped wedge interventional trial testing the superiority of the Clean Cut program over data collection alone as a means of improving perioperative outcomes following CS. It divides ten facilities into five separate clusters and randomizes the sequence of program intervention over a period of 10 months, with a lead-in baseline period and a post-implementation follow-up period for a total trial duration of 18 months.

### Study aims

The overall objective of the CLEAN-CS study is to assess the impact of a multimodal intervention, called Clean Cut, on surgical infections following CS. The primary and secondary aims are as follows:

#### Primary

1. To reduce postoperative infections in patients undergoing CS

#### Secondary

2. To reduce postoperative infections in patients undergoing other obstetric and gynecologic operations
3. To improve compliance with a core set of critical perioperative infection prevention and control practices that are essential to reducing infectious risks from surgical intervention
4. To reduce the need for reoperation in patients undergoing obstetric and gynecologic operations
5. To reduce the length of stay due to infectious and other complications for patients undergoing obstetric and gynecologic operations
6. To reduce mortality rates in mothers undergoing CS
7. To reduce mortality rates in women undergoing obstetric and gynecologic operations
8. To reduce mortality rates in neonates delivered by CS

#### Ancillary

9. To assess facility readiness for and capacities to engage in quality improvement programs in surgery, and evaluate whether such an assessment can be used to inform support of implementation

### Study endpoints

 These aims will be captured through collection of the following patient and facility endpoints:

#### Primary

1. Surgical infections following cesarean delivery: Number of patients undergoing cesarean delivery diagnosed with postoperative infection in hospital or up to 30 days post surgery; measured by change pre and post intervention

#### Secondary

2. Surgical infections following obstetric and gynecologic operations: Number of patients undergoing obstetric and gynecologic operations diagnosed with postoperative infection in hospital or up to 30 days post surgery; measured by change pre and post intervention
3. Compliance with infection prevention practices: Number of patients undergoing obstetric and gynecologic surgery whose operation adhered to each of the six perioperative infection prevention and control practices defined by the Clean Cut program; measured by change pre and post intervention
4. Reoperation following obstetric and gynecologic surgery: Number of patients requiring reoperation or return to the operating theatre prior to discharge following obstetric and gynecologic surgery; measured by change pre- and post-intervention
5. Length of stay: Mean and median length of stay, in days, following obstetric and gynecologic surgery; measured by change pre- and post-intervention
6. Postoperative maternal mortality: Number of mothers who die in hospital or up to 30 days following CS; measured by change pre- and post-intervention
7. Postoperative mortality: Number of women who die in hospital or up to 30 days following obstetric and gynecologic surgery; measured by change pre- and post-intervention
8. Neonatal mortality: Number of newborn/fetal deaths prior to discharge of mother following cesarean delivery; measured by change pre- and post-intervention

#### Ancillary

9. Atlas/MKA Facility Readiness Toolkit scores: Evaluation of facility characteristics—including assessments of Commitment and Motivation, Ability to Implement, Internal Culture, Clinical Team Functionality, and Knowledge and Ability to engage in quality improvement programs in surgery—as measured by the Atlas/MKA Facility Readiness Toolkit

These aims and endpoints are listed in Table 1, along with data sources (described in more detail below) and whether the specific data points are considered part of quality improvement or research.

**Table 1:** Aims and endpoints

Aim type	Aim	Endpoint	Data source	Data collection is part of quality improvement (QI) or research?
Primary	1. Infection reduction in CS	Surgical infection	In hospital: Medical records, direct observation	QI
			At 30 days: Phone call	Research
Secondary	2. Improved compliance with infection prevention practices	Compliance	Direct observation during surgery	QI
	3. Infection reduction in Ob/Gyn cases	Surgical infection	In hospital: Medical records, direct observation	QI
			At 30 days: Phone call	Research
	4. Reduction in unplanned reoperation	Reoperation	Direct observation, medical records, theatre logs	QI
	5. Reduced length of stay	Length of stay	Direct observation, medical records	QI
	6. Reduced maternal postoperative mortality	Maternal mortality	In hospital: Medical records, direct observation	QI
			At 30 days: Phone call	Research
	7. Reduced postoperative mortality	Postoperative mortality	In hospital: Medical records, direct observation	QI
			At 30 days: Phone call	Research
	8. Reduced neonatal mortality	Neonatal mortality	In hospital: mortality at time of discharge	QI
Ancillary	9. Assessment of readiness for QI generally and CLEAN-CS in particular	Facility readiness score	Interviews, surveys	Research

### Intervention

Clean Cut is a program developed by Lifebox that focuses on improving compliance with six critical perioperative infection prevention standards: (1) appropriate skin preparation of the surgeon’s hands and the surgical site; (2) maintenance of the sterile field by ensuring the integrity and sterility of surgical gowns, drapes, and gloves; (3) confirmation of instrument sterility; (4) appropriate antibiotic administration; (5) complete swab counts; and (6) routine use of the WHO Surgical Safety Checklist

Clean Cut is implemented in *five phases*:

- Phase 1: Identification of a Clean Cut team to include members from all perioperative disciplines: Ob/Gyn, surgery, nursing, anesthesia, QI personnel and operating room (OR) management;
- Phase 2: Establishment of a data collection system to track surgical infections and outcomes and understand context and facility readiness;
- Phase 3: Modification and implementation of the WHO Surgical Safety Checklist to fit local practices and process mapping the six perioperative standards;
- Phase 4: Data feedback to connect baseline data with process maps, coupled with site-specific action plans for improvement; and
- Phase 5: Targeted training, workshops, and refresher courses delivered by local providers, coupled with a transition to hospital management for sustaining the program.

For this study, Clean Cut has been adapted specifically for obstetric and gynecological operations and will be implemented in ten maternity hospitals/departments in Ethiopia in order to reduce infections and other complications for women undergoing CS and other obstetric and gynecologic operations. Clean Cut has been designed with sustainability at its core - the strategy emphasizes teamwork and collective leadership to identify and address critical gaps in perioperative safety processes. It develops sustainable facility-level and operating team management practices and embeds critical routines into the workflow of surgical teams. In addition, it imparts a classic quality improvement methodology that, anecdotally, has spread to other departments and services in hospitals where we have worked. As outlined above, a core function of the program involves supporting hospital teams to develop and commit to a sustainability plan to monitor and ensure continued adherence to best practices, something frequently agreed to but not necessarily achieved. Finally, it is “adaptive” as there are numerous opportunities for failures in perioperative infection control and prevention practices; Clean Cut leverages local knowledge and team dynamics to improve compliance through an iterative process that is developed and executed by the local teams. Solutions may be unique to a specific facility and may not be applicable, or replicable, in a different setting. The strategy and approach of Clean Cut, however, are based on classic quality improvement techniques, are replicable, and are introduced and implemented in each facility in a similar way.

Lifebox has partnered with the Ethiopian Society of Obstetricians and Gynecologists (ESOG) and Ariadne Labs in Boston, USA, to implement the CLEAN-CS trial. The study will test core elements of the improvement aspects of Clean Cut, namely phases 3, 4, and 5 outlined above, by randomly assigning the start of Phase 3 as part of the stepped wedge intervention testing strategy.

### Program evidence

Lifebox developed Clean Cut as a multimodal, adaptive, checklist-based improvement program following an extensive consultation with providers and practitioners from around the world who identified surgical infections as a major source of preventable surgical morbidity and mortality. The program was introduced in Ethiopia, initially in conjunction with the Surgical Society of Ethiopia and then through partnerships with the FMOH [11]. Clean Cut was first evaluated at Jimma University Specialized Hospital in 2016, with the pilot work extending to five initial hospitals where we prospectively collected compliance data from 2213 operations (374 during baseline assessment and 1839 following implementation of process improvements) in 2202 patients with follow up in 2159 patients (98.0%). At baseline, perioperative teams complied with an average of 2.9 of the six critical perioperative infection prevention standards; following process improvement changes, compliance rose to 4.5 (*p*<0.001). The relative risk of surgical infections following Clean Cut implementation was 0.65 (95% CI 0.43–0.99; *p*=0.043). Improved compliance with standards reduced the risk of postoperative infection by 46% (RR 0.54 for adherence score 3–6 vs 0–2; 95% CI 0.30*-*0.97; *p*=0.038).

Since then the program has been implemented in 11 hospitals and, as of late 2021, has benefitted an estimated 80,000 patients. In our preliminary, unpublished analyses of 8 hospitals with completed data, we have prospectively collected compliance data from 2905 operations, of which 1133 (39%) were CS or hysterectomy, with an average monthly volume of 27 obstetric operations per facility. Of the total, 2692 patients (92.7%) had outcomes follow-up, with 1075 (40%) being obstetric patients. After program implementation, significant improvements were seen in each of the infection prevention areas: instrument decontamination, gown and drape integrity, surgical skin antisepsis, gauze counting, antibiotic administration, and use of the surgical safety checklist. At baseline, unadjusted SSI rate was 11.3% overall and 12.4% for the obstetric group; after Clean Cut implementation, the SSI rate fell to 6.2% overall and 6.6% in the obstetric group (a 47% decrease).

Some studies have described the impact of quality improvement (QI) programs on SSI reduction in low-resource settings, and the use of process measures instead of or in addition to patient outcomes is important to identify actionable interventions [14]. However, in a literature review of 354 studies on surgical QI in LMICs, only 11% used process measures as a metric [15]. The African Surgical Unit-based Safety Program, a multimodal surgical infection prevention program implemented in five hospitals in Sub-Saharan Africa, focused on improving perioperative process measures including preoperative bathing, hair removal, skin and hand preparation, antibiotic administration, and OR traffic, showed significant improvements and an associated reduction in SSI from 8.0 to 3.8% [16]. This intervention leveraged local providers to implement evidence-based guidelines. However, it did not focus on checklist use, sponge counts, instrument sterility, or gown and drape reprocessing, all of which have been noted as major gaps in our setting [17].

The Clean Cut program has demonstrated significantly improved compliance with critical infection prevention standards and reduced postoperative infections without requiring major investments in new infrastructure or resources. Like similar programs, uncontrolled aspects of implementation limit specific attribution to Clean Cut itself. Many factors may have influenced success, including staff characteristics, prior strong QI programs, and engaged hospital administration. Our sample size was small, particularly during the baseline period, which limits robust comparisons of outcomes between groups before and after implementation. A larger, more rigorous trial such as a cluster-randomized stepped wedge design will validate this approach and determine whether Clean Cut can be replicated and scaled in different hospitals, countries, and settings.

### Study sites and facility eligibility

Study sites will consist of ten hospitals in Ethiopia that provide maternal surgical services. Five of these hospitals will be university teaching or referral hospitals (aka specialized or referral hospitals), and five will be regional, district, or smaller community hospitals (aka general or regional hospitals). These hospitals have been selected by the Ethiopian Society of Obstetricians and Gynecologists (ESOG) based on the following factors:

1. They perform no less than an average of 30 cesarean deliveries per month over 3 months
2. They have not received intensive quality improvement training by partner NGOs within the last 2 months
3. There is no plan to deliver intensive quality improvement training by partner NGOs within the next 6 months
4. They have the capacity to follow patients on the wards and contact patients by phone at 30 days postoperatively
5. They are accessible by the study team
6. They accept the national IRB approval and do not require additional local IRB review

### Patient eligibility

Any patient of any age undergoing obstetric and gynecologic surgery at any time in one of the targeted operating theatres is eligible for inclusion; there will be no exclusion criteria.

### Participants

As obstetric and gynecologic operations are typically undertaken in separate, dedicated operating theatres, we will focus our prospective observations on patients admitted to these theatres. Any patient undergoing surgery at any time in one of the targeted operating theatres is eligible for inclusion; there will be no exclusion criteria. Enrollment will occur at the time of observation and will include various times (day and night) and days of the week (weekdays and weekends). As the standards being implemented are not in dispute and are considered critical for ensuring antisepsis and sterility, patient informed consent will not be obtained. While our focus will be on cesarean delivery, any obstetric or gynecological operation will be included, with the inclusion of other operations in these populations (such as appendectomy for appendicitis that is found incidentally or misdiagnosed as ovarian torsion, for example). There will be no age range limit.

We will also interview key hospital personnel to understand the context of each facility, its experience with quality improvement initiatives, and the perceived importance of this work to patient safety, patient care, and the work routine. These interviewees will be recruited from the implementation teams involved in Clean Cut. We will also administer surveys in conjunction with Ariadne Labs/MKA, a partner in this work, to understand the context, perceptions, and priorities of the various institutions, and how the CLEAN-CS team can support implementation at the time of intervention (phases 3, 4, and 5)

### Site randomization

Each selected hospital will be distributed into two groups based on the type of facility: university teaching and referral hospitals in one group, and regional, district, and community hospitals in another. One hospital in each group will be paired to create five clusters; these pairings will be purposive as district and referral hospitals in Ethiopia typically have long-standing relationships which will facilitate implementation at the cluster level and prevent inadvertent crossover of the intervention prior to randomization. The sequence of implementation for each of the five clusters will be established by the Lifebox team using computer-based randomization (https://www.randomizer.org/). Sites will be informed of the timing of their intervention no earlier than one month prior to implementation training.

### Study design

The impact of the CS-tailored Clean Cut program will be tested through a cluster-randomized stepped-wedge study design in five clusters (10 hospitals) over the course of 18 months followed by complete data analysis and interpretation. The selected hospitals will start by identifying a clinical lead and data collectors who will collect inpatient and outpatient outcomes on all patients undergoing CS prior to implementation as well as compliance information on the six standards of Clean Cut (phases 1 and 2 of Clean Cut outlined above). Following a lead-in period of 5 months, one cluster at a time will begin implementing phase 3 of Clean Cut at 2-month intervals over the course of 10 months. The intervention is initiated by assembling a multidisciplinary improvement team, undergoing team training on WHO Surgical Safety Checklist use and implementation, and reviewing compliance information about intraoperative safety practices. The initiation of phase 3 will also involve creating facility-specific process maps of each critical perioperative practice. Once these process maps and compliance data are complete, usually after 2–4 weeks, the team establishes an adaptive, facility-driven improvement plan based on process gaps and barriers to best practice (phase 4). This is initially facilitated by Lifebox, but may also involve teams that have already implemented Clean Cut in their facilities. They identify improvement opportunities by reviewing process mapping data, compliance shortfalls, and their bespoke surgical safety checklist, and craft an improvement plan aimed at removing barriers to compliance. The team will review their hospital-specific compliance data and patient outcomes on a monthly basis for the remainder of the study. The study will conclude at each site after 18 months of data collection

### Timing and mechanism of intervention

The program is adaptive and relies on a multimodal approach to improving compliance with critical safety practices. Initial trainings take place at the outset of the work to coordinate data collector training and orient some of the key team members to the work (phases 1 and 2). The first Clean Cut implementation training (phase 3) will occur following at least 3 months of baseline data collection and will be introduced based on randomization. As noted above, this will specifically include a multidisciplinary perioperative improvement team to create facility-specific process maps of each critical perioperative practice. Once these process maps are complete, usually after 2–4 weeks, the team establishes an adaptive, facility-driven improvement plan based on process gaps and barriers to best practice (phases 4 and 5).

At study initiation and over the course of implementation, the Atlas/MKA Context Assessment Tools will be used to understand facility-level readiness for implementing quality improvement programs in surgery [18]. The tools assess the following readiness domains: Commitment and Motivation, Ability to Implement, Internal Culture, Clinical Team Functionality, and Knowledge and Ability to do the Practice Change using Likert scale, self-administered surveys, and a discussion guide for facilitated readiness conversations. The intent is to use the findings to support successful implementation.

Once implementation teams have identified opportunities for improvement based on the local process mapping exercise, specific gaps are targeted based on local solutions for improvement. Almost always a training and education program is included in the work to inform health care workers about changes to routines and practices, and to reinforce standards. Several specific training programs have been developed by Lifebox focusing on checklist implementation strategies, antibiotic stewardship, instrument reprocessing practices [19], and teamwork and communication techniques using nontechnical skills strategies. Methods for improving sponge counting during surgery have also been identified and introduced [20].

Lifebox will provide technical assistance for all training and workshops associated with the program, such as Surgical Safety Checklist workshops targeted towards CS, Safe Instrument Reprocessing workshops, and refresher training on surgical infection prevention standards. Lifebox will also provide technical assistance for staff training on Clean Cut implementation, data collection mechanisms, and data entry and visualization using our data capture platform (described below). Programmatic support includes coordination and management of the hospital-designated implementation teams, data quality assurance, and coordination of partners within Ethiopia. ESOG will guide site selection, organize local trainings, identify local trainers from among its membership, and disseminate results through its extensive networks. ESOG will also provide technical and institutional support for obstetric clinicians at study sites. Ariadne Labs will provide trial design and statistical support during conception, data analysis, and interpretation, as well as support using the Atlas/MKA tools and interpreting their findings.

### Outcomes assessment

We will assess changes in compliance with the critical perioperative safety practices over time and reductions in complications including surgical infections within 30 days, need for reoperation, and death before and after implementation.

### Power calculations and sample size

**Fig. 1:**
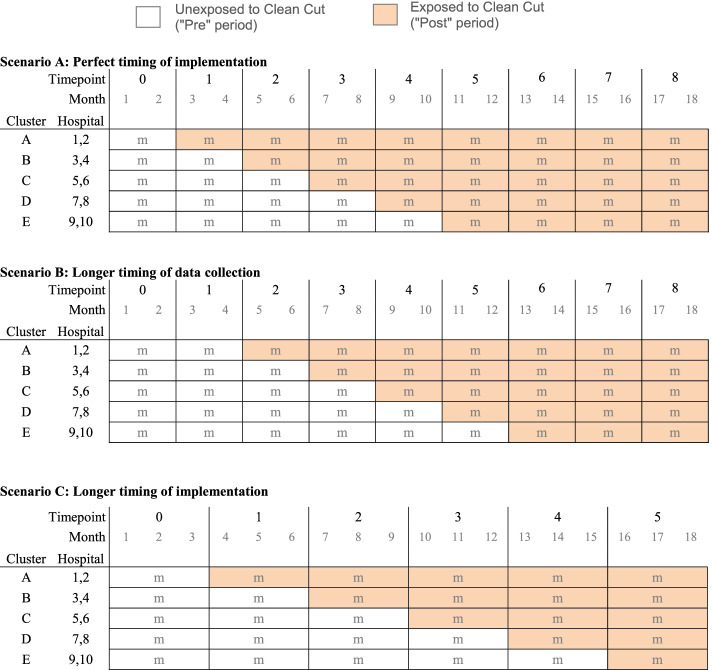
Explanatory stepped wedge designs

Our prior work and review of the literature indicate a baseline SSI incidence of 12% following CS. Given our past work has reduced SSI by 35%, we assume an effect size of Clean Cut resulting in a 25% absolute reduction in SSI (from 12 to 9%). We expect that the preintervention sample will match the postintervention sample in size and general demographic characteristics. We have recruited 10 hospitals that will be paired up into five clusters of two hospitals each, thus providing five steps in the stepped wedge design, as shown in Fig. 1. As we do not have specific information on the interhospital characteristics or outcomes, we assume an intracluster correlation (ICC) of 0.1.

We follow the notation of Hemming and Taljaard [21] in Fig. 1. In both scenarios, each row represents one cohort, which is a pair of hospitals that form a cluster. Each column represents one time period, and as in any stepped wedge, there is one more time period than steps in the wedge. Lower case “m” represents the sample size contributed by each cluster in each timepoint. The shading displays the different start dates for Clean Cut over the study period.

Scenario A is the planned timing for the steps. Note that in this scenario, the sample sizes in the exposed “post” group will be 30m, which is larger than in the unexposed “pre” group size of 15m. However, due to field conditions, the start dates for implementation are unlikely to be as quick as in Scenario A. In Scenario B, delays in data collection would result in more evenly matched pre/post cohort sizes, with 20m in the unexposed “pre” group versus 25m in the “post” group—especially if the intervention occurs in the middle of the time periods. In Scenario C, delays in implementation between each cluster also results in two comparison groups of nearly equal size. We expect that the actual stepped wedge as implemented in the study will be irregular, but roughly between scenarios B and C. For the purposes of power calculations, we will assume that the sample will be split roughly evenly between the unexposed and exposed conditions.

Our power calculations proceeded in two steps. First, we calculated the sample size needed for a pre-post comparison of two proportions in dependent samples (using the SAS function proc power, paired freq, text = McNemar). We used these as inputs:

- Alpha = 5%
- Power = 80%
- Proportion 1 = Unexposed proportion = 12% (infection rates)
- Proportion 2 = Exposed proportion = A range from 8% to 9.5% (infection rates)

We then calculated the design effect of the clustered stepped wedge, again following the method of Hemming and Taljaard [21]. The design effect is a multiplier for the sample size calculated in the first step, showing how much larger the sample should be to account for clustering while maintaining the same alpha and power to detect the given difference [22]. The design effect is calculated as:

$${\mathrm{DE}}_{\mathrm{SW}}=\left(t+1\right)\frac{1+\rho \left( tm+m-1\right)}{1+\rho \left( tm/2+m-1\right)}\times \frac{3\left(1-\rho \right)}{2\left(t-1/t\right)}$$

where:

DE_SW_ = design effect multiplier for the stepped wedge design

*t* = the number of clusters = 5 (Thus the number of timepoints = t+1 = 6)

*m* = the sample collected in each cluster per timepoint (noting that the timepoint may include different numbers of months, as shown in Fig. 1).

*ρ*= the intracluster correlation (ICC) between patients in the same cluster.

**Fig. 2:**
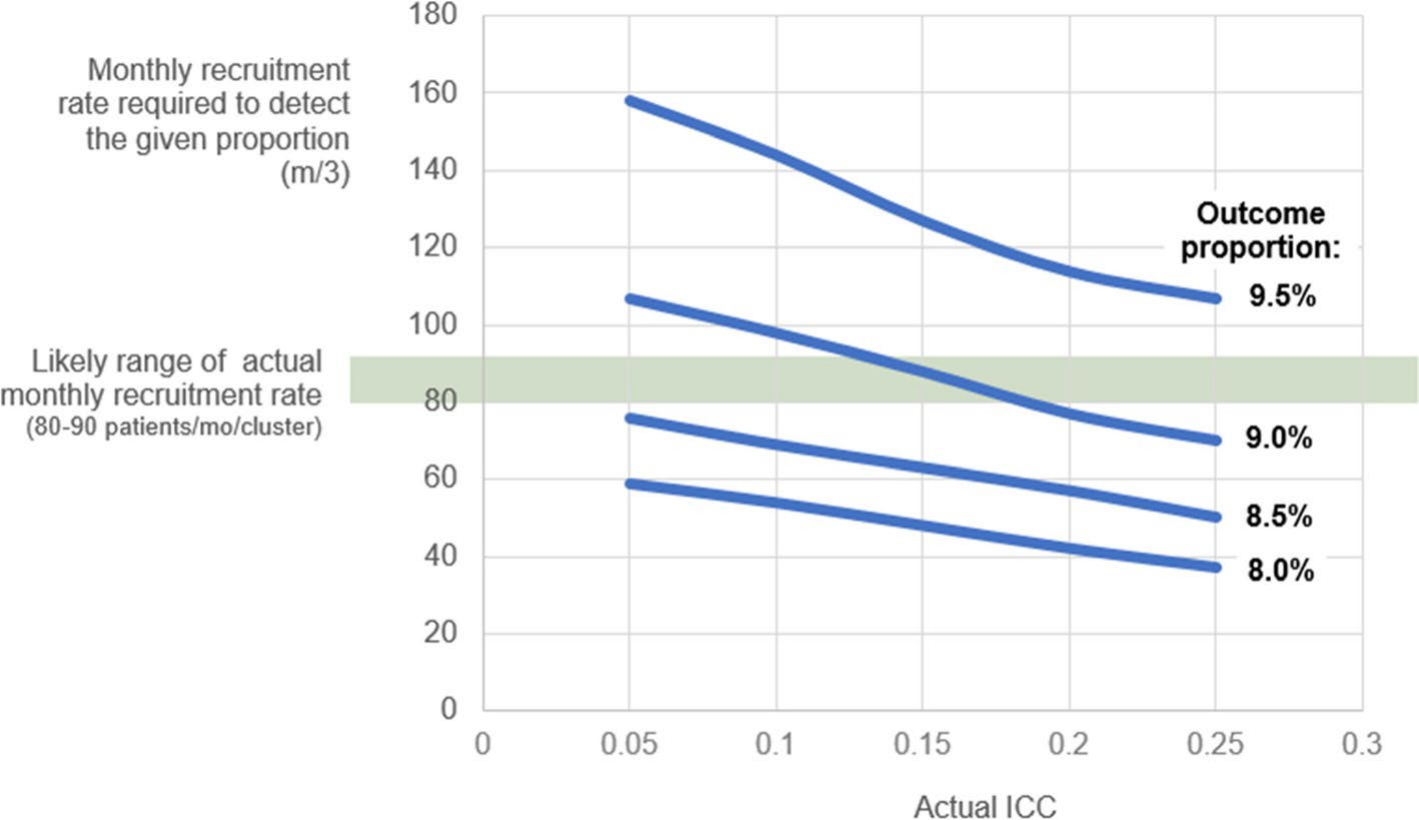
Monthly recruitment rates needed to detect selected outcome proportions

Given patient volume at each cluster and the eligibility of all CS surgical patients, we expect to be able to recruit 80 to 90 patients per month per cluster. Figure 2 shows the monthly study recruitment rates needed at a range of ICCs and a range of expected proportions for the outcome. (These results reflect Scenario B from Fig. 1). The green area in Fig. 2 highlights the expected recruitment rate, and the lines on this graph show the monthly sample sizes that would be needed at various ICCs in order to detect the difference between the given outcome (infection rate) and baseline outcome (starting infection rate of 12%), while maintaining alpha error and power. For example, if the actual ICC is 0.15 and the proportion of CS patients with SSI under Clean Cut is 9%, then we will have sufficient power to detect that 3-point difference from 12% since we will be recruiting in the required range of sample size. Note that we will have more than enough power to detect areas below the green line, so if the effect of Clean Cut lowers the SSI rate even more, we will be able to comfortably detect it. If the outcome proportion is 9.5%, however, we will not likely have sufficient power to detect that difference from 12%.

With recruitment of 80–90 patients per cluster per month over 18 months, we expect the final sample to include 7200 to 8100 patients, which will be sufficient to detect our expected difference within a reasonable range of ICCs [22].

### Analysis

We will conduct pre/post analyses using the statistical approach recommended by Hemming [21] with our planned assessment controlling for risk factors and other demographic and procedure variables listed below that are known to affect infection rates. We will compare patient demographics pre and post intervention as well to evaluate the overall matching of patients in each part of the study. We will evaluate compliance individually and in an all-or-none manner as previously described in our Clean Cut pilot work. We will undertake a planned subanalysis of patients observed early during baseline (first 2 months) and compare them to patients undergoing surgery during the final stage of the study (last 2 months) after implementation of the program has had time to take effect to assess primary and secondary outcomes.

As we are interested not only in the primary outcome of surgical infection but also in process improvements and the implementation strategies and support that can lead to effective compliance with best practices, we will not conduct any interim analyses. The purpose of an interim analysis is to assess the study for benefit, futility, or harm. We believe that neither benefit nor futility would be readily observable at an interim analysis given the implementation strategy and approach; furthermore, even if we were able to assess benefit or futility we would want to continue the trial to fully study the mechanisms of implementation. Regarding an assessment of harm, if such an outcome were noted we believe this would most likely be due to detection bias with improving ability to capture complications as the study progressed, and we would want to let the study play out to mitigate this detection bias over time.

### Data components

#### Compliance with standards

In this stepped wedge study, the institutions will be assessed for their baseline and post implementation compliance of the six critical perioperative infection prevention standards noted previously. In addition, we will study the impact on outcomes of patients after the intervention. This will be done by both inpatient and outpatient surveillance of the primary and secondary outcomes set by this study both at the baseline time and after the intervention.

#### Inpatient surveillance

Ongoing, systematic collection and analysis of data on patients who have CS or any gynecological procedure while they are still admitted in the wards. The data will be captured from the first postoperative day until the day of discharge. The data include the condition of the surgical wound for any sign of infection; any other site of infection; the type and duration of antibiotics; need for and indication of reoperation; length of stay; and maternal and/or neonatal mortality and cause of death.

#### Outpatient surveillance

Ongoing, systematic collection and analysis of data on patients who have CS or any obstetric or gynecological procedure after they are discharged until the 30th day post surgery. This follow-up will typically occur through phone follow-up. The data includes the condition of the surgical wound for any sign of infection as redness at the wound site, wound discharge, wound dehiscence, wound dressing offered at local health center; any other known or recognized infection; readmission; reoperation and indication if known; maternal and/or neonatal mortality and cause of death if known.

#### Outcome variables

As noted in Table 1, outcomes will include patient-level endpoints as well as process compliance endpoints, including postoperative infections within 30 days, compliance to each of the six standards of surgical site infection prevention and control that comprise Clean Cut, unplanned reoperation, length of hospital stay, maternal and postoperative deaths within 30 days of surgery, and neonatal status (alive or dead) at discharge of the mother.

#### Demographic variables

These include basic demographic characteristics of the patient: age, gestational age, gravida/parity; pregnancy comorbidities such as hypertension, pre-eclampsia, gestational diabetes, obesity, malnourishment; other comorbidities such as HIV and anemia; timing of onset of labor, and timing of rupture of membranes.

#### Procedure variables

These include information of the operation itself, including emergency vs elective case status, date and time of incision, duration of operation, wound classification (according to the CDC definition), estimated blood loss, intraoperative complications or mishaps, procedure name, indication for surgery, and whether meconium is present (if CS).

### Data collection and quality assurance

#### Data collector training

Data collectors will be trained on the data collection process including definitions of each variable and the appropriate process for observing and recording data elements onto the forms. Data collector training may be separated by cohort to tailor the training to nurses or other data collectors who will collect the different “phases” of data—intraoperative, inpatient ward follow-up and outpatient phone-call follow-up. Data entry personnel will also be trained how to use the DHIS2 platform to create patient encounters and enter data including enrollment, intraoperative, inpatient and outpatient data. They will also be trained on access and interpretation of the “follow-up” dashboards, where patient record completeness can be tracked. A data dictionary will be available to all data collectors and entry staff.

#### Data capture

Sites will aim to collect data on at least 50 patients per facility (100 per cluster); based on our prior experience with loss to follow-up and incomplete data capture, we anticipate capturing complete data on 80-90 patients per cluster per month. Data collection will occur on a predetermined auditing schedule or on a 100% enrollment basis, to be determined by the hospital-based Clean Cut team based on monthly case volumes. Minimum enrollment numbers have accounted for facility volume of cesarean sections. If using an auditing basis care will be taken to distribute the weekend/weekdays, shift times, and days per week to eliminate as much bias as possible from the types of cases being enrolled.

Patients will be enrolled in the Clean Cut program in the intraoperative phase, with their first encounter being their index operation. Once enrolled, OR data collectors will communicate and handover enrolled patients to the appropriate wards. Inpatient data will be collected by the ‘ward’ data collectors on a daily basis, through chart review and direct observation of the patient’s surgical wound. Patients will be followed, data collected, and entered on a daily basis until discharge from the hospital or 30 days following surgery. At discharge, the ‘ward’ data collectors will communicate and handover information to the responsible data collectors for the outpatient follow-up encounter. A phone call will be placed at 30 days following surgery to gather outpatient data on mortality, clinical follow-up and signs of surgical infection. All data collected on hard copy paper forms will be transferred to the data entry personnel at time intervals to be determined by the hospital team, ideally on at least a daily basis. Data will be entered on a daily basis by the data entry personnel, with attention paid to entering all available data from hard copies into the DHIS2 system as soon as possible.

#### Data review, monitoring, and quality assurance

Site coordinator or lead data manager at each hospital will review data entries every 2–3 days, flagging those with missing or incomplete data and communicating with data entry personnel and/or data collectors with queries. A data quality officer will review all hospital data on a weekly basis in a meeting with each hospital lead data manager. This review will also include time of day, day of week, and other observational inputs to ensure patient enrollment is representative of the spectrum of conditions treated by the hospital.

#### Missing data

Instances of missing inpatient or outpatient encounters will be tracked and when possible the missing data identified and entered. All missing data elements and missing encounters will be tracked by site coordinators overseen by data quality officer. Frequently missing data elements will be flagged and recurrent issues addressed with data collectors and data entry personnel.

### Trial and protocol management

#### Management and communications

Each study site will have a regular check-in with the trial management team in order to review recruitment targets and data quality and provide general bidirectional updates on progress. Site visits will occur on an ad hoc basis depending on challenges or specific issues or difficulties encountered. While randomization will be completed by and known to the study team leadership (TNM, MD, and TGW), all other members will be blinded to the cluster order. Unblinding of each intervention cluster will occur 2–4 weeks prior to intervention in order to allow for planning and organization at the facilities; the last cluster hospitals which will naturally know their order as the last interventional sites given the unblinding order.

#### Managing protocol challenges and potential disruptions

Given the current pandemic as well as potential civil unrest in Ethiopia, there is the potential for unanticipated challenges to implementing the protocol as described. While every attempt will be made to maintain participation of all sites, the loss of one or more sites is a distinct possibility. Recruitment of patients may also be affected by patient volumes, supply shortages, or facility closures. Given this, we will allow each site to collect up to 92 patients per month if they are able in case of the loss of a facility in a cluster. In the case where one facility in a cluster is no longer participating in the study, we will allow the other facility to increase data collection up to 150 cases per month, if feasible and the volume allows, and adjust our comparative calculations accordingly.

#### Human resources, roles, and responsibilities

Our core trials team consists of the authors of this manuscript. Tasks are allocated as follows:

- Coordination of trial sites: ST, TF, MA
- Data review, coordination, follow-up: HW, MA
- Protocol adherence: TNM, MD, MA, TGW
- Implementation support: TNM, ST, NG, HW, NS, MA, SB
- Consent for interviews: ST, TF, MA, HW
- Trial oversight and supervision: TNM, MD, TGW

Members of this group meet weekly to review progress, discuss challenges, raise and address concerns, and ensure plans for trial delivery. Initial discussions focused on timing, organization, coordination, and site staffing and training issues. Subsequent meetings have focused on data quality, compliance challenges, coordination of site leads and data collectors, transparency, and qualitative work and interviews with site personnel. We have also assembled an advisory group comprised of three external members not involved in the trial who provide additional feedback and input into the approach and strategies for engagement. This group also evaluates the progress of the trial with respect to recruitment and overall implementation successes and challenges. It is comprised of a public health expert and professor of Ob/Gyn from South Africa, a professor of Ob/Gyn from Rwanda, and a pediatrician and child health specialist working for the Ethiopian Federal Ministry of Health.

Each facility has a designated site lead and 2–4 data collectors who complete data forms. Site leads recruit and organize the local data collectors, arrange meetings, and identify and develop a local team that can coordinate improvements following intervention. ESOG coordinates all aspects of local agreements and payment for work in conjunction with Lifebox. All active personnel are eligible for inclusion in the authorship under a study investigators group authorship.

### Data management, compliance, and security

#### Data storage

Paper data collection forms will be stored in a secure, locked location on-site at study hospitals and made accessible to study personnel only. Patient-level data will be entered into the DHIS2 system using password-protected, encrypted, hospital-specific accounts granted to study personnel. Readiness data will be entered into a secure Qualtrics form.

#### Reporting

Our study will be reported according to guidance extending the CONSORT 2010 statement on reporting stepped wedge cluster randomized trials [23].

#### Protection of human subjects

The study protocol was reviewed and approved by the Armauer Hansen Research Institute (AHRI/ALERT) Ethics Review Committee, one of the nationally accredited ethical boards, on 8^th^ February 2021. Following approval with AHRI, it was forwarded to the National Research Ethics Review Committee which oversees national trials and received approval in this secondary review on 9 June 2021, reference 04/246/965/21. This approval was renewed in May 2022. The ethics review committees approved a waiver of informed consent for patients undergoing surgery given the quality improvement nature of the work as stated in the submitted proposal that was reviewed. The investigators have obtained a letter of support from the Federal Ministry of Health and have an agreement in place with each institution, thus further ethical approval was not sought from the individual institutions. Our interviews with and surveys of providers and team members will help inform the work and the support needed by facilities and hospitals; this was included in the IRB review, and informed consent will be obtained during the interview and survey processes. The lead investigators (TNM, MDF, TGW) will ensure that this trial is conducted in accordance with relevant regulations and with Good Clinical Practice. Patient data will be kept confidential throughout the study and stored on encrypted servers and password-protected devices.

#### Trial registration

The trial has been registered with ClinicalTrials.gov, identifier NCT04812522 on 23rd March 2021, and with the Pan-African Clinical Trials Registry, identifier PACTR202108717887402 on 24th August 2021.

## Discussion

Our CLEAN-CS trial using a stepped wedge cluster randomized protocol will allow us to evaluate the effectiveness, implementation, impact, and barriers and facilitators to implementing Clean Cut to improve compliance with widely accepted perioperative infection prevention standards. The choice of a cluster-randomized stepped wedge design is particularly appropriate for evaluating the implementation of this type of surgical patient safety program. The infection prevention standards targeted by the program are not in doubt, yet compliance is frequently difficult. Solutions are available and may be recognized by individuals, but entire teams with a shared vision to drive implementation are often lacking. The trial embeds the Clean Cut surgical infection prevention intervention into obstetric care settings; the program has been successfully piloted but requires assessment using a more rigorous methodology. While there is a strong emphasis on quality improvement, the research component seeks to understand the qualities, characteristics, and resources needed to implement the program, the magnitude of the effect on process compliance and resultant outcomes of care, and the effect-specific activities of process improvement have on compliance with critical standards of perioperative infection prevention and control.

Past criticism of such work, including of the SSC itself, frequently focuses on the observation that the act of data collection can result in improved care, the so-called Hawthorne Effect. In CLEAN-CS we seek in part to evaluate whether data collection alone is enough to drive improvements in care or whether, and to what degree, a more formalized improvement program using process mapping and teamwork is critical for enhanced compliance with care standards.

The choice of a trial design was made because no hospital was willing to forego the intervention nor did we feel that we had equipoise to withhold the intervention given our prior findings of a powerful improvement in compliance with standards and reductions in infections. However, given our limited ability to scale quickly across 10 facilities in different regions of the country, we were in a strong position to deliver the Clean Cut program to all hospitals over a 1–2-year timeframe and chose to randomize the order of implementation despite the logistical challenge of such a trial.

There are substantial challenges to this work, including the fraught social and political environment in Ethiopia, pandemic travel restrictions, and a limited budget. Ethiopia is currently suffering a civil war, with fighting in the northern regions (Tigray and parts of Amhara). We chose facilities in Addis Ababa and to the East, West, and South; we purposefully avoided sites that were or might be directly affected by the conflict. Travel restrictions due to COVID-19 also limit our ability to conduct direct training of trainers, site visits, and in-person educational sessions. We have followed a conservative travel policy and only recently, at the direction of the FMOH, eased travel restrictions and facility visits. Since the internet and network connectivity is intermittent and frequently subject to interruptions, hosting virtual training sessions frequently fails to communicate all necessary information, thus requiring time-intensive one-on-one training and distance mentoring for data collectors and site leads. Finally, several data collectors and other site-specific personnel expressed dissatisfaction with the remuneration agreement. As our budget is limited, this has required ongoing discussion with a potential loss of sites or turnover of trained staff. We work with each site lead to ensure the staff understand the effort, agree to the terms of payment and scope of work, experience the challenges and rewards of participating in a clinical trial, and appreciate the value of this quality improvement effort.

### Trial status

This trial is currently recruiting at selected sites in Ethiopia. Data collection commenced 24th August 2021 and is anticipated to continue for 18 months.

## Supplementary Information

**Additional file 1.**

## Data Availability

Data will be made available upon reasonable request in anonymized form no early than 3 months after publication of a final manuscript. Following completion of the trial and publication, data will be made available in anonymized form upon reasonable request.
